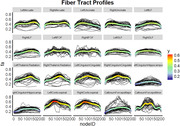# Fiber tract profiles of community dwelling older adults with LATE‐NC and associations with postmortem TDP‐43

**DOI:** 10.1002/alz.094979

**Published:** 2025-01-09

**Authors:** Ashley Heywood, Julie A. Schneider, David A. Bennett, Konstantinos Arfanakis, Lei Wang

**Affiliations:** ^1^ Northwestern University, Chicago, IL USA; ^2^ Rush University, Chicago, IL USA; ^3^ Rush Alzheimer’s Disease Center, Rush University Medical Center, Chicago, IL USA; ^4^ Rush University Medical Center, Chicago, IL USA; ^5^ Ohio State University Wexner Medical Center, Columbus, OH USA

## Abstract

**Background:**

TAR DNA‐binding protein 43 (TDP‐43), has been shown to be involved in various neurodegenerative disorders involving axonal damage including ALS, FTLD, and LATE. Studying the relationships between postmortem TDP‐43 and antemortem white matter (WM) structural integrity can allow for a better understanding of underlying neural mechanisms of the disease. Measures of white matter integrity assume fiber bundles to maintain similar characteristics along the length of the tract, however, advanced computational research has identified that white matter integrity varies in stereotyped patterns along the tract.

**Methods:**

In‐vivo diffusion‐weighted images were gathered on a 1.5T scanner from subjects from the Religious Orders Study and the Rush Memory and Aging Project. Tractography was conducted for each subject using Mrtrix3 and Automated Fiber Quantification (AFQ) was used to calculate fractional anisotropy (FA) along fiber tracts for 20 major fiber bundles. A semi‐quantitative rating of TDP‐43 severity was assessed in 5 brain regions. We utilized regression models to relate postmortem disease and antemortem FA at 100 nodes along each fiber tract while controlling for coexisting Alzheimer’s disease pathology and demographics.

**Results:**

The 63 subjects were 91.2 (SD = 6.2) [range: 71.7‐103.6] years old at death. Thirty‐eight percent had TDP‐43 pathology and 36% had cognitive changes before death. Overall, fiber tract profiles revealed variation of FA along each fiber bundle. Results measuring the relationship to disease revealed a significant negative relationship (FWE corrected‐p<0.05) between postmortem TDP‐43 and FA at unique portions of the left hippocampal cingulum.

**Conclusion:**

Findings support current literature indicating white matter integrity does not follow a uniform pattern along fiber tracts in older adults. Pathology relationships reveal unique portions of tracts which have unique relationships to TDP‐43. Understanding the relationship between disease and these vulnerable regions will be critical in elucidating the effect of TDP‐43 on white matter integrity leading to cognitive decline.